# Validity of predictive equations for 24-h urinary sodium excretion at the population and individual levels among Chinese adults aged 18–69 years

**DOI:** 10.1038/s41598-021-00513-1

**Published:** 2021-11-17

**Authors:** Xiaofu Du, Le Fang, Jing Guo, Xiangyu Chen, Shuoci Su, Jie Zhang

**Affiliations:** 1 Department of Chronic Disease Prevention and Control, Zhejiang Provincial Center for Disease Control and Prevention, No. 3399 Binsheng Road, Hangzhou, 310051 China; 2 Department of Public Health, Institute of Environmental Health, Zhejiang University School of Medicine, No. 866 Yuhangtang Road, Hangzhou, 310011 China; 3 Bloomberg School of Public Health, Johns Hopkins University, No. 2024 E. Monument Street, Baltimore, 21287 USA

**Keywords:** Nutrition, Public health, Risk factors

## Abstract

Spot urine (SU) collection is a convenient method commonly used for sodium estimation, but its validity in predicting 24-h urinary sodium (24-hUNa) excretion has not been thoroughly evaluated among the general population. The aim of this study was to comprehensively assess the validity of eight existing methods in predicting 24-hUNa excretion by using SU samples among Chinese adults. We analyzed 1424 representative individuals aged 18 to 69 years. We compared the measured and estimated measurements of 24-hUNa at the population level by examining bias, the correlation, intraclass correlation coefficients (ICCs), receiver operating characteristic (ROC) curves and Bland–Altman plots and analyzed the relative and absolute differences and misclassification at the individual level. The bias for all methods was significant (all *p* < 0.001), among which the smallest bias was − 7.9 mmol for the Toft formula and the largest bias was − 53.8 mmol for the Mage formula. Correlation coefficients were all less than 0.380, all formulas exhibited an area under the ROC curve below 0.683, and the Bland–Altman plots indicated slightly high dispersion of the estimation biases at higher sodium levels regardless of the formula. The proportions of relative differences > 40% for the eight methods were all over one-third, the proportions of absolute differences > 51.3 mmol/24 h (3 g/day NaCl) were all over 40%, and the misclassification rates (7, 10, and 13 g/day NaCl as cutoff points) were all over 65%. Caution remains due to poor validity between estimated and actual measurements when using the eight formulas to obtain a plausible estimation for surveillance of the Chinese population sodium excretion, and the results do not support the application of SU to estimate sodium intake at the individual level due to its poor performance with respect to classification.

## Introduction

Increased blood pressure (BP) has become a major risk factor for the global burden of disease. The prevalence of hypertension in China has increased to 23.2%, which means that approximately 244.5 million Chinese adult individuals have hypertension^1^, and subsequently, stroke and heart disease lead to death and DALYs at the national level^2,3^. It was suggested that high sodium is one main key risk factor for hypertension. The previous prevalence of daily salt intake in China is 10.5 g/day^4^, which is far greater than the amount (less than 5 g/day) recommended by the World Health Organization (WHO)^5^. A study of the global burden of disease found that three million deaths were attributable to a high-sodium diet^6^. Therefore, reducing sodium intake in the general population, which has been considered a cost-effective action and recommended as one of the top three priority actions to tackle the global crisis of noncommunicable disease by the WHO, should be maintained and expanded to save more lives, prevent diseases and avoid costs^7–10^. Both to achieve goals such as a 30% relative reduction in mean population salt intake by 2025 set by WHO^11^ or 20% by 2030 set by the Chinese Central Government and given that the determination of salt intake is considered decisive in the individualization of therapy in patients with, for example, renal hypertension and resistant hypertension^12^, the highest priority and most essential step is to ensure that the mean population or individual salt intake can be correctly assessed and continuously monitored.

There are currently several methods to assess salt intake, comprising food frequency questionnaires, dietary recall or records, and measurement of 24-hUNa excretion^13^. In general, 24-hUNa excretion has been regarded as the ‘gold standard’ and the most valid method for evaluating daily salt intake^14,15^. However, because of the associated high participant burden and high cost and given the large within-individual variability in salt consumption as a result of single 24-h urine assessment and incomplete urine in practice^16,17^, alternative methods of 24-hUNa excretion testing are consistently being studied. Although several studies have been developed to establish formulas that may allow the estimation of individual 24-hUNa excretion using SU specimens and have been validated in external populations and areas, such as the Kawasaki formula^18^, Tanaka formula^19^, INTERSALT with or without potassium formulas (INTERSALT1 and INTERSALT2, respectively)^15^, Toft formula^20^, Whitton formula^21^, Mage formula^22^, and Sun formula^23,24^, the suitability of these formulas to estimate salt intake in individuals and populations is still controversial. There is a certain difference between the estimated value of the prediction formula and the actual measurement value at the individual level, and the large differences in the characteristics of the population among different regions result in the limitation of the extrapolation of the prediction formula. Until now, whether these formulas are reasonable predictive methods for the Chinese population has remained an unresolved issue. Consequently, the validity of predictive equations needs further research among the larger Chinese population with multiple and comprehensive statistical methodologies. In this study, for over 1500 representative individuals from which we collected both specimens of 24-h urine and morning fasting urine, we plan to evaluate the validity of different formulas both at the population level and at the individual level in China. This study conducted a comprehensive and informative statistical analysis. We tested all hypotheses before formula verification and explored the conditions for the promotion and use of the prediction formula as while tried to find reasons for the bias.

## Methods

### Study population

Cross-sectional survey data were analyzed from the baseline survey of the Salt Reduction and Hypertension Prevention Project (SRHPP) in Zhejiang Province of China in 2017. The participant flow chart is shown in Supplementary Fig. 1. In short, through the stratified multistage random sampling method, 75 administrative villages or neighborhood committees in 5 counties or cities were randomly selected to carry out the project in Zhejiang Province to achieve balance and representativeness. A roster was established in each selected village or neighborhood committee, and qualified residents were randomly selected and mobilized face-to-face to participate in the project. In the end, 1512 residents formed a representative sample at the provincial level to provide informed consent and successfully retained 24 h of urine to participate in the project. The method of sample size calculation and sample selection is described in detail elsewhere^25^. The project was approved by the Ethical Review Committee of Zhejiang Provincial Center for Disease Control and Prevention, and written informed consent was obtained from all participants. All methods consisting of questionnaire surveys, physical examinations and biological specimen testing were performed in accordance with the relevant guidelines and regulations.

Trained technicians were invited to ask all interviewees to participate in a closed survey. Information about the demographic characteristics of participants was collected through questionnaire surveys. Every individual sampled underwent a physical examination to obtain BP, weight, height, etc. Body mass index (BMI) was calculated from the formula of weight (kg)/height (m)^2^. According to international measurement methods and quality control specifications, rachial systolic BP and diastolic BP measurements were performed in triplicate with the participant seated for at least 5 min for rest. Measurements were recorded at least one minute apart using a validated automatic electronic sphygmomanometer (HEM-7071, Omron Corp., Japan) with an appropriately sized cuff, and the average value of the three measurement results was taken.

### Urine collection and laboratory testing

The 24-h urine was used to calculate the sodium excretion of the participants within 24 h. During the investigation, a leaflet with explanations along with the necessary equipment (3 L and 50 mL standard urine collection container) was provided to 1512 participants, and 24-h urine and morning urine collection procedures were explained^26^, i.e., urinate on waking on the first day, collect approximately 4 mL of midstream urine into a 5 mL container as the morning urine sample and then collect all urine including that voided first thing after waking the next day into a 3 L container as the 24-h urine sample. At the urine collection site, the 24-h urine volume was measured, and the start and end times of urine collection were recorded. Aliquots (5 mL) of the two kinds of urine samples were taken and shipped using a cold chain to a central lab (KingMed Diagnostics Laboratory Inc., Hangzhou, China) for sodium, potassium and creatinine assays, with the remainder discarded. An ion selective electrode method was used for sodium and potassium analysis (C16000, Abbott Corp., America), and a picric acid method was used for creatinine analysis (C501, Roche Cob as Corp., Switzerland). Twenty-four-hour urine specimens (measured 24-hUNa excretion) were calculated by multiplying the sodium concentration by the corresponding volume of the sample.

Urine specimens were considered incomplete if the length of collection time was less than 22 h, total urine volume was less than 500 mL, or 24-h urinary creatinine excretion was ± 2 standard deviations outside of the sex-specific mean^27,28^.

### Estimation of 24-hUNa excretion from SU specimens

We used eight previously published equations to predict 24-hUNa excretion from spot urinary sodium concentrations (Table 1). These equations were developed through the approaches of direct regression or rationale of equivalent sodium-to-creatinine ratio. However, regardless of the formula, they are all based on age, sex, height, weight, and urinary electrolytes in SU specimens.

**Table 1:** Equations for 24-h urinary sodium excretion based on single spot urine samples.

Method	Published time	Study population	Age range (years)	Urine specimen	Predictive formula (mmol/24-h)	Applicable population
Kawasaki	1992	Japanese Men and women (n = 159)	20–79	SMU	Male: 16.3 × [Na_su_/(Cr_su_ × 10) × (7.39 × height + 15.12 × weight − 12.63 × age − 79.9)]^0.5^ Female: 16.3 × [Na_su_/(Cr_su_ × 10) × (5.09 × height + 8.58 × weight − 4.72 × age − 74.5)]^0.5^	General population
Tanaka	2002	Japanese Men and women (n = 591)	20–59	Casual spot urine	21.98 × [Na_su_/(Cr_su_ × 10) × (16.14 × height + 14.89 × weight-2.04 × age − 2244.45)]^0.392^	General population
INTERSALT1	2012	Western (North American and European) Men (n = 2841), Women (n = 2852)	20–59	Casual spot urine	Male: (0.46 × Na_su_ + 25.46) − 2.75 × Cr_su_(mmol/L) − 0.13 × K_su_ + 4.10 × BMI + 0.26 × Age Female: (0.34 × Na_su_ + 5.07) − 2.16 × Cr_su_(mmol/L) − 0.09 × K_su_ + 2.39 × BMI + 2.35 × Age − 0.03 × Age^2^	General population
INTERSALT2	2012	Western (North American and European) Men (n = 2841), Women (n = 2852)	20–59	Casual spot urine	Male: (0.45 × Na_su_ + 23.51) − 3.09 × Cr_su_(mmol/L) + 4.16 × BMI + 0.22 × Age Female: (0.33 × Na_su_ + 3.74) − 2.44 × Cr_su_(mmol/L) + 2.42 × BMI + 2.34 × Age − 0.03 × Age^2^	General population
Toft	2012	Danish Men and women (n = 473)	18–65	Casual spot urine	Male: 33.56 × [Na_su_/(Cr_su_ × 10) × (− 7.54 × Age + 14.15 × weight + 3.48 × height + 423.15)]^0.345^ Female: 52.65 × [Na_su_/(Cr_su_ × 10) × (− 6.13 × Age + 9.97 × weight + 2.45 × height + 342.73)]^0.196^	General population
Whitton	2016	Southeast Asian (Singapore residents of Chinese, Malay, and Indian ethnicity) Men and women (n = 144)	18–79	SMU	Male: 88.66 + 0.55 × Na_su_ − 1.34 × Cr_su_(mmol/L) − 1.05 × K_su_ − 0.87 × Age + 2.10 × BMI + 39.30 Female: 88.66 + 0.55 × Na_su_ − 1.34 × Cr_su_(mmol/L) − 1.05 × K_su_-0.87 × Age + 2.10 × BMI	General population
Mage	2008	Used to estimate urine pesticide and chemical exposure with NHANES urine specimens	NA	Casual spot urine	Male: Na_su_/(Cr_su_ × 10) × [0.00179 × (140 − Age) × weight^1.5^ × height^0.5^] Female: Na_su_/(Cr_su_ × 10) × [0.00163 × (140 − Age) × weight^1.5^ × height^0.5^]	General population
Sun	2015	Chinese Men and women with hypertension (n = 334)	26–76	SMU	Male: 0.218 × [e^(5.961–0.009×Age + 0.005×height + 0.013×weight)^] × (Na_su_/Cr_su_/10)^0.344^ Female: 0.180 × [e^(5.824–0.010×Age + 0.007×height + 0.010×weight)^] × (Na_su_/Cr_su_/10)^0.302^	Hypertension patients, excluding taking antihypertension medicine, secondary hypertension, chronic kidney disease

The units of concentration for Na_su_ and K_su_ were all mmol/L; unless otherwise specified, the units of Cr_su_ were mg/dl. The units of weight and height were kg and cm.

*SMU* second morning urine, *Na*_*su*_ Spot urinary sodium, *K*_*su*_ Spot urinary potassium, *Cr*_*su*_ Spot urinary creatinine.

### Statistical analysis

The baseline characteristics of the participants were summarized as count (percentage) proportions and means (SD, standard deviation). Independent samples *T*-tests were used to compare the demographic and health characteristics among participants across gender categories. At the population level, we assessed the quality of estimations for 24-hUNa excretion by examining the bias relative to the observed measurements (estimated values minus measured excretions). A paired samples *T*-test was performed to test whether the mean bias was statistically significant. Pearson’s correlation coefficient and ICC were used to assess the associations between estimated 24-hUNa excretion from eight estimation formulas and measured values. Moreover, we computed the sensitivity, specificity, cutoff value and probability of area under curve (AUC) with ROC to assess the capacity of each formula to discriminate individuals with arbitrarily considered low sodium intake (< 3000 mg/24-h) from those with high sodium intake (≥ 3000 mg/24-h). Bland–Altman plots with linear trends and 95% CIs for the mean bias were used to compare the agreement of the estimated sodium intakes with the measured values. We computed the expected value of difference (i.e., bias) from regressing differences between the estimated and the measured sodium over their average (difference = *b0* + *b1*average) and then regressed the absolute value of residuals of this model over the average from these estimated and measured sodium values (residuals = *c0* + *c1*average). *b0* + *b1*average and *b0* + *b1*average ± 2.46(*c0* + *c1*average) were computed to represent the linear trend for the mean bias and the 95% limits of agreement, respectively^29^. At the individual level, we computed the relative and absolute difference between estimated and measured 24-hUNa excretion, according to the formulas [(estimated − measured)/measured] and (estimated − measured), respectively. Then, estimated and measured 24-hUNa excretion were converted into salt intake and divided into 4 categories with a 3-g difference in salt intake (< 7, 7–9.99, 10–12.99, ≥ 13 g/24-h) due to cutoff points of categorization close to but more intuitive than the quartile distribution^30^. Finally, the fractions of individuals classified into different groups correctly and subsequent misclassification rates for each formula were calculated. All data were analyzed using SPSS 26.0 (IBM Corp., Armonk, New York, USA) and R 3.6.3. Statistical significance was defined as *p* < 0.05 with two-sided tests.

## Results

Of participants selected for 24-h urine collection, 1424 (94.2% of 1512) returned a complete specimen. The baseline characteristics of the participants in our study are summarized by sex in Table 2. A total of 1424 individuals were included in the present analysis. The mean age of the individuals was 46.7 ± 14.1 years, and males accounted for 48.9%. The mean 24-h urine volume and measured 24-hUNa excretion were 1443.0 mL and 165.7 ± 71.5 mmol/24 h (equivalent to a calculated salt intake of 9.7 ± 4.2 g/day), respectively.

**Table 2:** Characteristics of the 1424 participants according to gender.

	All (n = 1424)	Male (n = 697)	Female (n = 727)	*p* value^a^
**Age (years)**	46.7 ± 14.1	46.7 ± 14.4	46.7 ± 13.7	0.924
18–29 (n, %)	240 (16.9%)	122 (17.5%)	118 (16.2%)	
30–39 (n, %)	266 (18.7%)	136 (19.5%)	130 (17.9%)	
40–49 (n, %)	256 (18.0%)	120 (17.2%)	136 (18.7%)	
50–59 (n, %)	320 (22.5%)	143 (20.5%)	177 (24.3%)	
60–69 (n, %)	342 (24.0%)	176 (25.3%)	166 (22.8%)	
Weight (kg)	62.9 ± 10.8	67.6 ± 10.4	58.4 ± 9.2	< 0.001
Height (cm)	161.6 ± 8.1	167.1 ± 6.5	156.4 ± 5.6	< 0.001
BMI (kg/m^2^)	24.0 ± 3.3	24.2 ± 3.2	23.9 ± 3.4	0.057
Systolic BP (mmHg)	130.0 ± 19.6	133.7 ± 18.2	126.5 ± 20.3	< 0.001
Diastolic BP (mmHg)	80.0 ± 11.0	82.2 ± 10.7	77.9 ± 10.8	< 0.001
**Spot urine**				
Sodium concentration (mmol/L)	125.1 ± 49.7	125.6 ± 48.9	124.5 ± 50.5	0.675
Potassium concentration (mmol/L)	32.5 ± 17.0	32.6 ± 17.7	32.4 ± 16.4	0.865
Creatinine concentration (mmol/L)	12.5 ± 6.5	14.2 ± 6.7	10.9 ± 5.9	< 0.001
**24-h urine**				
24-h urine volume (mL)	1443.0 ± 441.5	1476.8 ± 461.9	1410.5 ± 418.8	0.005
Sodium excretion (mmol/24-h)	165.7 ± 71.5	172.6 ± 74.5	159.1 ± 67.9	< 0.001
Potassium excretion (mmol/24-h)	37.2 ± 16.8	36.2 ± 18.0	38.1 ± 15.4	0.030
Creatinine excretion (mmol/24-h)	9.5 ± 3.9	10.9 ± 4.2	8.2 ± 3.1	< 0.001

^a^Differences between males and females were tested by independent samples t-tests.

### Accuracy at the population level

The mean bias between estimated and measured 24-hUNa excretion is presented in Table 3. Among the eight predicted formulas, the mean bias for the Toft formula, which exhibited the smallest difference, was − 7.9 mmol (95% CI − 11.5, − 4.2 mmol/24-h). The Mage formula with the largest mean bias was − 53.8 mmol/24 h. The bias of estimated and measured 24-hUNa excretion for all methods was significant (all *p* < 0.001). Correlation analysis of 24-hUNa excretion estimated from SU specimens by the eight methods indicated that the estimated 24-hUNa excretion was weakly positively correlated with the measured 24-hUNa excretion. The correlation coefficients (between 0.300 and 0.380) were low (all *p* < 0.001), among which the highest Pearson’s r was 0.380 for Sun and the lowest Pearson’s r was 0.303 for Toft. Similarly, the ICCs (between 0.22 and 0.32) were all low (all *p* < 0.001), among which the highest was 0.32 for Kawasaki and the lowest was 0.22 for INTERSALT2.

**Table 3:** Comparison between measured and estimated sodium excretion using eight formulas for 24-hUNa excretion (n = 1424).

Method	Bias				Estimation accuracy^d^				Agreement with Bland–Altman plot	
	24-hourUNa (mmol/24-h)	Mean Bias (mmol/24-h, 95% CI)^a^	r_p_^b^	ICC (95% CI)^c^	AUC^e^	Sensitivity^f^	Specificity^g^	Cutoff value (mmol/24-h)^h^	95% of difference (mmol/24-h)^i^	Probability that estimated value exist within mean ± 1.96 SD^j^
Measured	165.7 ± 71.5									
Kawasaki	183.8 ± 56.7	18.1 (14.3 to 22.0)	0.345	0.32 (0.27 to 0.38)	0.664	49.3%	75.1%	187.2	291.7	94.8% (1350/1424)
Tanaka	142.9 ± 35.6	− 22.7 (− 26.3 to − 19.1)	0.327	0.24 (0.17 to 0.31)	0.657	55.9%	67.7%	140.8	269.4	94.9% (1352/1424)
INTERSALT1	133.6 ± 33.7	− 32.1 (− 35.6 to − 28.5)	0.340	0.23 (0.12 to 0.32)	0.664	65.6%	60.2%	124.0	266.3	94.9% (1351/1424)
INTERSALT2	130.3 ± 33.5	− 35.4 (− 38.9 to − 31.9)	0.342	0.22 (0.10 to 0.32)	0.665	63.2%	62.5%	122.2	266.0	95.2% (1356/1424)
Toft	157.8 ± 39.1	− 7.9 (− 11.5 to − 4.2)	0.303	0.25 (0.20 to 0.30)	0.633	65.4%	55.6%	141.0	275.8	95.2% (1355/1424)
Whitton	135.6 ± 38.2	− 30.1 (− 33.6 to − 26.6)	0.370	0.27 (0.16 to 0.36)	0.676	55.1%	73.9%	140.8	264.7	95.6% (1362/1424)
Mage	111.9 ± 77.4	− 53.8 (− 58.3 to − 49.4)	0.343	0.27 (0.09 to 0.41)	0.679	57.1%	69.1%	93.4	335.2	94.9% (1351/1424)
Sun	121.2 ± 35.7	− 44.5 (− 48.0 to − 41.0)	0.380	0.23 (0.06 to 0.37)	0.683	61.3%	68.3%	114.6	261.7	94.8% (1350/1424)

*24-hUNa* 24-h urinary sodium, *r*_*p*_ Pearson correlation coefficient, *ICC* intraclass correlation coefficients, *AUC* area under the curve.

^a^Mean bias all *p* < 0.001.

^b^Person correlation all *p* < 0.001.

^c^The values of single measures were used, and all *p* < 0.001.

^d^The results of accuracy of prediction come from ROC curve.

^e^Area under the ROC curve for predicting 24-hUNa in individuals whose sodium intake was ≥ 3000 mg/24-h (AUC), and the *p* value represents the significance value to refute the null hypothesis of being nondiscriminatory, and all *p* < 0.001.

^f^The sensitivity is defined as the proportion of individuals whose measured 24-h urinary sodium ≥ 3000 mg with the estimated amount ≥ 3000 mg at the cutoff value.

^g^The specificity is defined as the proportion of individuals whose measured 24-h urinary sodium < 3000 mg with the estimated amount < 3000 mg at the cutoff value.

^h^The cutoff value is defined as the corresponding detection value at the maximum value of [sensitivity − (1 − specificity)].

^i^Ninety-five percent of the difference is the width between the upper limit and lower limit in the Bland–Altman plot.

^j^The probability that the difference between the measured and estimated values existed within − 1.96 SD and + 1.96 SD of the mean value in the Bland–Altman plot.

**Figure 1:**
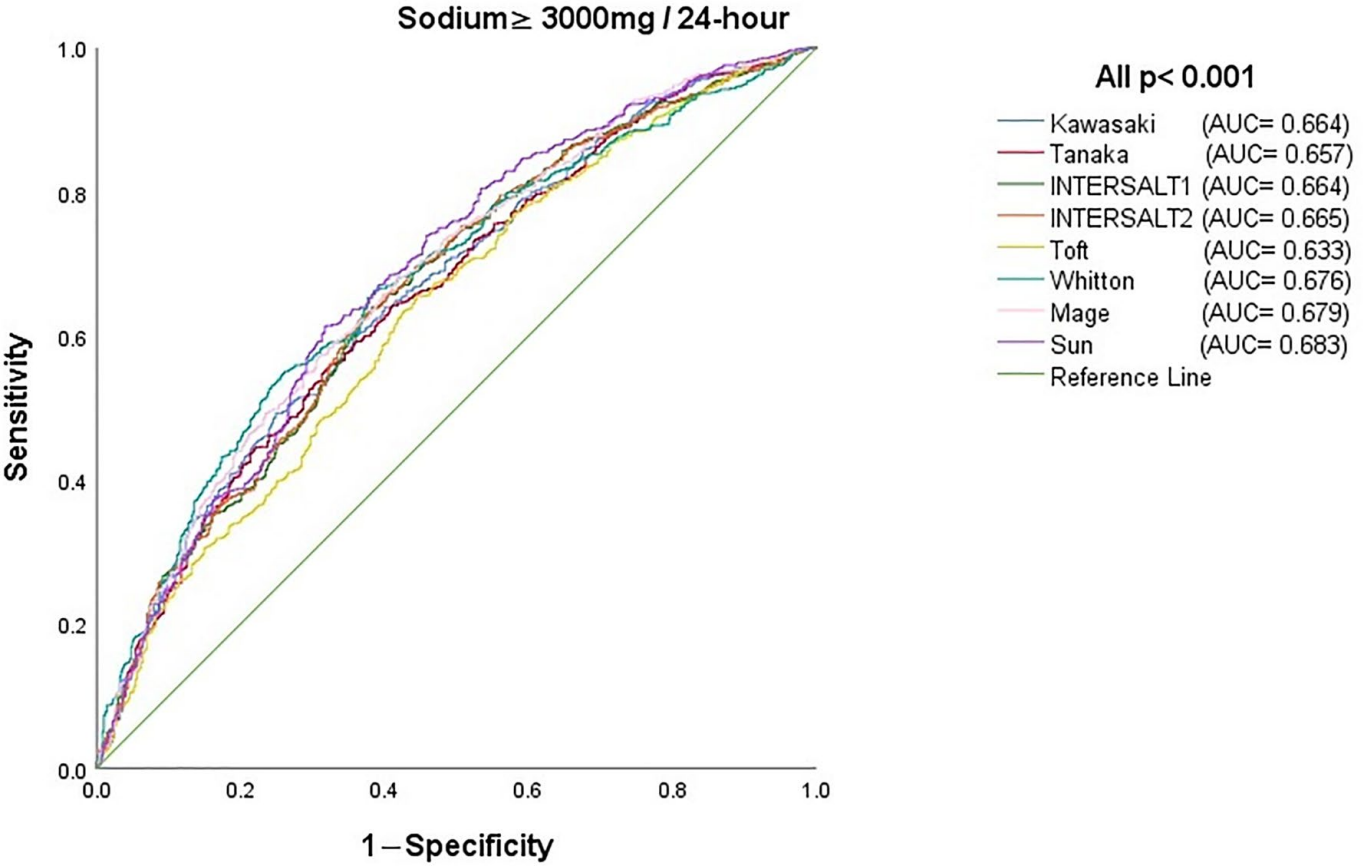
Area under the ROC curve for predicting the 24-h urinary sodium level among individuals whose sodium level was more than 3000 mg/24 h.

The ROC curves indicated that even though each formula could distinguish individuals who have a high sodium intake from those with a low intake (all *p* < 0.001), all formulas showed inadequate discriminatory capacity presenting an AUC below 0.683 (Fig. 1). The highest sensitivity of formulas to estimate the measured 24-h urinary sodium ≥ 3000 mg/24-h with the estimated amount ≥ 3000 mg/24-h was 65.6% for the INTERSALT1 formula. However, the specificity was the highest at 75.1% for the Kawasaki formula. Regarding the best corresponding cutoff value of Youden’s index, the largest and smallest were 187.2 mmol/24 h and 93.4 mmol/24 h for the Kawasaki and Mage formulas, respectively, and other cutoff values were relatively moderate. In addition, we compared the AUCs of eight prediction formulas in pairs, and the results showed that the AUC of Toft was smaller than that of the others, and those of Mage and Sun were larger than those of one or two other methods (Supplementary Table 1).

**Figure 2:**
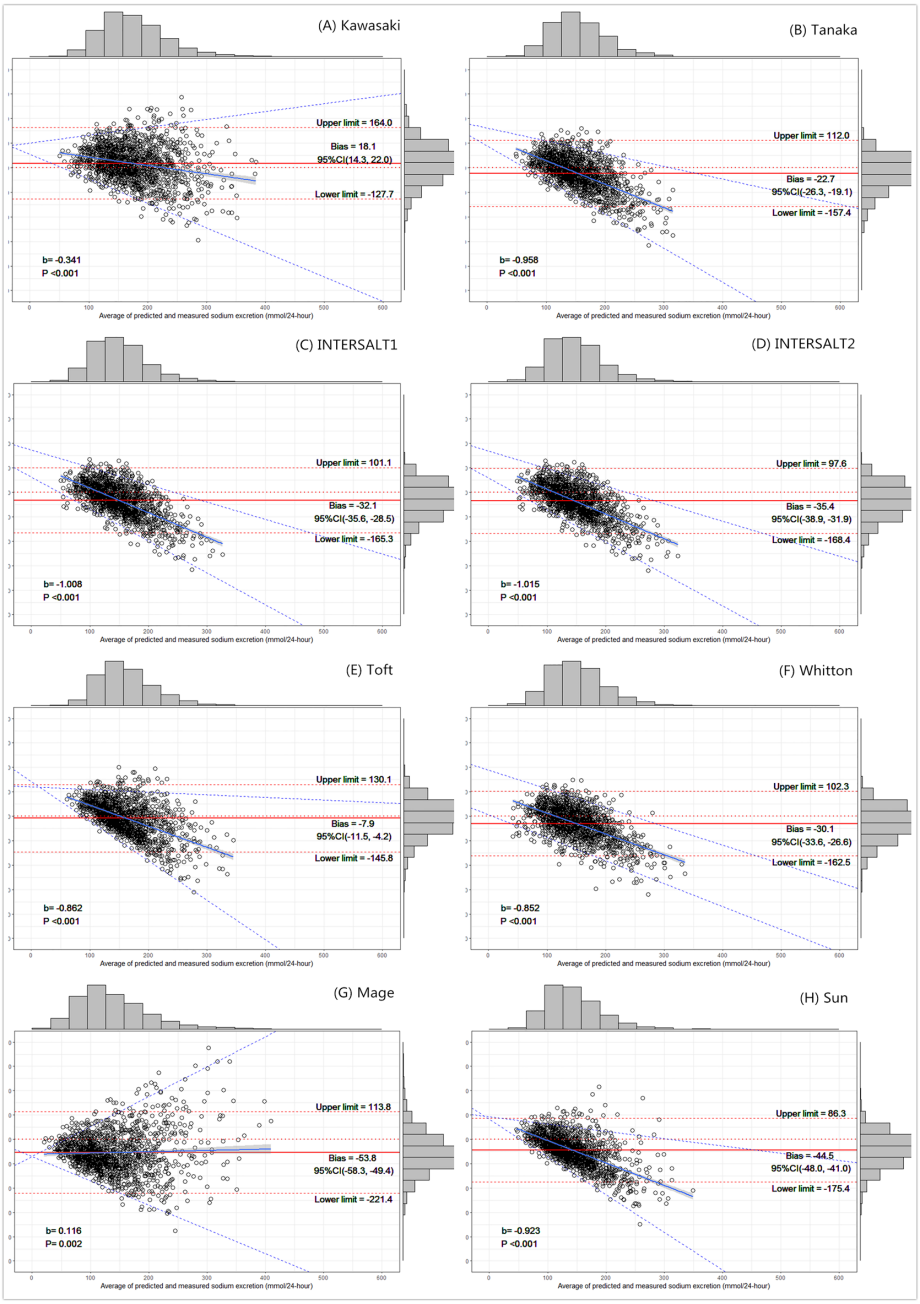
Bland–Altman plots of the estimated and measured 24-h urinary sodium excretion based on the Kawasaki Equation (**A**), Tanaka Equation (**B**), INTERSALT1 Equation (**C**), INTERSALT2 Equation (**D**), Toft Equation (**E**), Whitton Equation (**F**), Mage Equation (**G**), and Sun Equation (**H**).

As shown for the Bland–Altman plots of differences (estimated—measured) against the average measured and estimated 24-hUNa excretion for each formula (Fig. 2), in general, the lower levels of 24-hUNa excretion tend to have narrower 95% limits of agreement than the higher levels. The limits of agreement were widest with the Mage formula (335.2 mmol/24 h), followed by the Kawasaki formula (291.7 mmol/24 h). For all formulas, narrower limits of agreement facilitated the variability of the bias increase across increasing sodium levels; that is, the narrower the limit range was, the steeper the slope (the slope in the regression indicated the changes in bias per 1 mmol/24-h increase in the average estimated and measured 24-hUNa excretion). The regression showed that the trend of the bias was significant for each estimate by all formulas. The Mage and Kawasaki formulas presented a less extreme slope than the other formulas. Moreover, Bland–Altman plots further showed a negative trend of mean biases across the level of sodium excretion, with the only exception being the Mage formula; in other words, compared with the average measured value, seven estimates were systematically biased with overestimation at lower levels and underestimation at higher levels.

### Accuracy at the individual level

**Figure 3:**
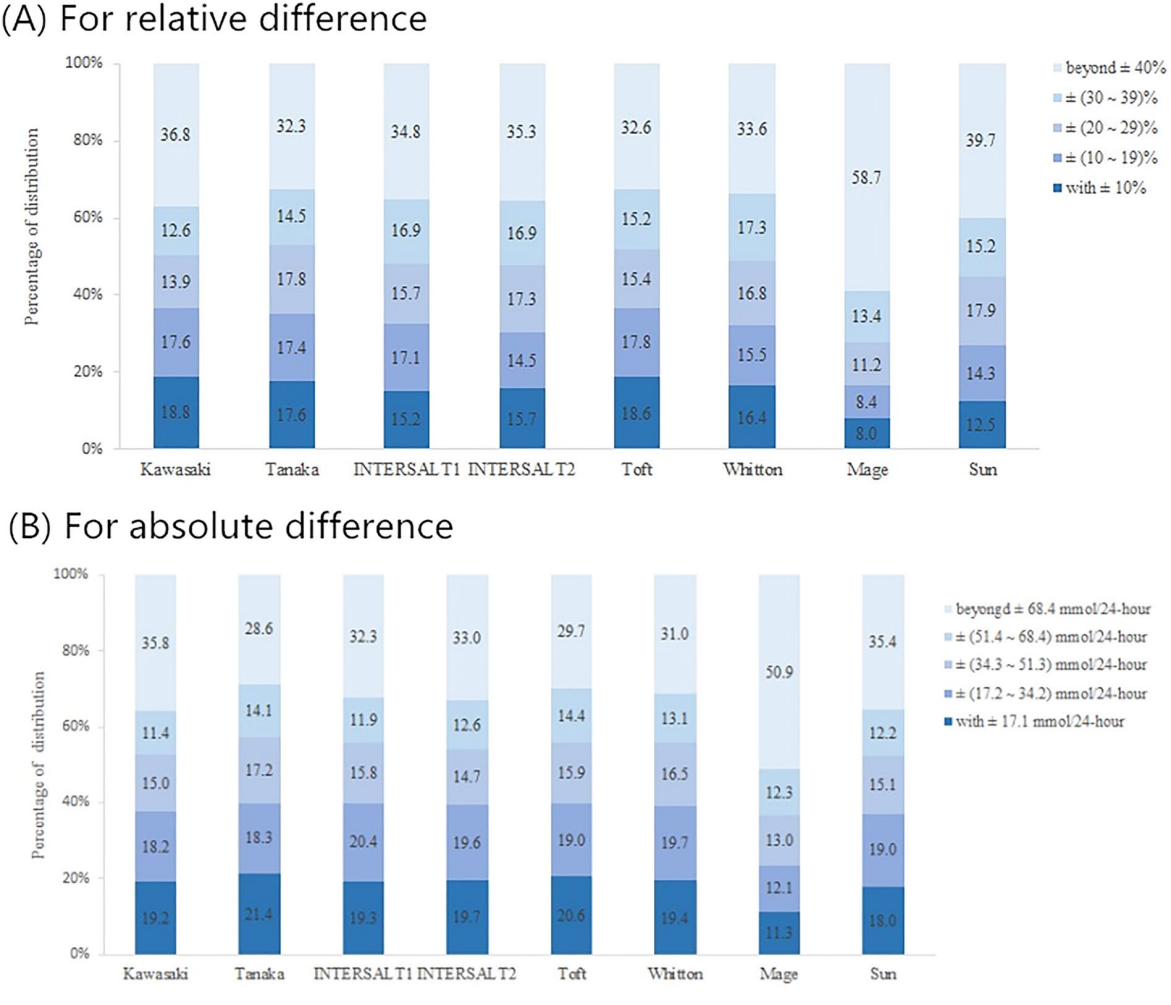
Relative and absolute difference distributions of 24-h urinary sodium excretion estimated based on eight equations. Relative difference (**A**) and absolute difference (**B**).

Taking the measured 24-hUNa excretion as a reference, the relative and absolute differences between estimated and measured 24-hUNa excretion are shown in Fig. 3. Among the comparison of relative differences between the eight formulas, the highest proportion of ‘within ± 10%’ was 18.8% for Kawasaki, and the lowest proportion was 8.0% for Mage. The proportions of ‘beyond ± 40%’ were all > 30%, among which the highest proportion was 58.7% for Mage. Among the comparison of absolute differences between the eight formulas, the highest proportion of ‘within 17.1 mmol/24-h (1 g/day NaCl)’ was 21.4% for Tanaka, and the lowest proportion was 11.3% for Mage. Furthermore, the proportions of absolute differences beyond 51.3 mmol/24 h (3 g/day NaCl) for the eight methods were all > 35%, among which the highest proportion was 50.9% for Mage.

We compared the classification group of individual salt intake according to the measured sodium excretion in comparison to the estimated sodium excretion. After classifying the estimated and measured 24-hUNa excretion into four categories (< 7, 7–9.99, 10–12.99, and ≥ 13 g/day NaCl), the misclassification rates of individual salt intake were all > 65%, with the highest 68.7% for Whitton and the lowest 65.6% for Kawasaki (Table 4). In addition, we found that each formula was suitable for evaluating sodium intake. For example, the highest correct classification rates of Kawasaki (44.1%) and Tanaka (52.2%) were mainly concentrated in the '7–9.99 g/24-h' group, while Mage (81.6%) and Sun (71.7%) showed the optimal correct classification effect in the '< 7 g/24-h' group. Compared with the mean measured sodium levels during the development of each formula, the closer the predicted sodium level is, the higher the prediction power (compare Table 4 with Supplementary Table 3).

**Table 4:** Misclassification of the eight predicted methods for individual salt intake level, n (%).

Method	Conversion of salt intake by 24-hUNa excretion				Total (n = 1424)
	< 7 g/24-h (n = 396)	7–9.99 g/24-h (n = 434)	10–12.99 g/24-h (n = 320)	≥ 13 g/24-h (n = 274)	
**Kawasaki**					
< 7 g/24-h	**73 (18.4)**	43 (10.0)	17 (5.3)	5 (1.8)	–
7–9.99 g/24-h	168 (42.4)	**190 (44.1)**	102 (32.0)	67 (24.5)	–
10–12.99 g/24-h	112 (28.3)	120 (27.8)	**112 (35.1)**	86 (31.5)	–
≥ 13 g/24-h	43 (10.9)	78 (18.1)	88 (27.6)	**115 (42.1)**	–
Misclassification	323 (81.6)	244 (56.2)	208 (65.0)	159 (58.0)	934 (65.6)
**Tanaka**					
< 7 g/24-h	**158 (39.9)**	133 (30.9)	57 (17.9)	38 (13.9)	–
7–9.99 g/24-h	195 (49.2)	**225 (52.2)**	183 (57.4)	138 (50.5)	–
10–12.99 g/24-h	39 (9.8)	63 (14.6)	**72 (22.6)**	81 (29.7)	–
≥ 13 g/24-h	4 (1.0)	10 (2.3)	7 (2.2)	**16 (5.9)**	–
Misclassification	238 (60.1)	209 (48.2)	248 (77.5)	258 (94.2)	953 (66.9)
**INTERSALT1**					
< 7 g/24-h	**203 (51.3)**	197 (45.7)	86 (27.0)	48 (17.6)	–
7–9.99 g/24-h	170 (42.9)	**184 (42.7)**	171 (53.6)	148 (54.2)	–
10–12.99 g/24-h	23 (5.8)	50 (11.6)	**61 (19.1)**	72 (26.4)	–
≥ 13 g/24-h	0 (0.0)	0 (0.0)	1 (0.3)	**5 (1.8)**	–
Misclassification	193 (48.7)	250 (57.6)	259 (80.9)	269 (98.2)	971 (68.2)
**INTERSALT2**					
< 7 g/24-h	**224 (56.6)**	214 (49.7)	99 (31.0)	58 (21.2)	–
7–9.99 g/24-h	151 (38.1)	**174 (40.4)**	166 (52.0)	145 (53.1)	–
10–12.99 g/24-h	21 (5.3)	43 (10.0)	**53 (16.6)**	67 (24.5)	–
≥ 13 g/24-h	0 (0.0)	0 (0.0)	1 (0.3)	**3 (1.1)**	–
Misclassification	172 (43.4)	260 (59.9)	267 (83.4)	271 (98.9)	970 (68.1)
**Toft**					
< 7 g/24-h	**76 (19.2)**	61 (14.2)	21 (6.6)	7 (2.6)	–
7–9.99 g/24-h	243 (61.4)	**264 (61.3)**	185 (58.0)	140 (51.3)	–
10–12.99 g/24-h	63 (15.9)	80 (18.6)	**90 (28.2)**	79 (28.9)	–
≥ 13 g/24-h	14 (3.5)	26 (6.0)	23 (7.2)	**47 (17.2)**	–
Misclassification	320 (80.8)	170 (39.2)	230 (71.9)	227 (82.8)	947 (66.5)
**Whitton**					
< 7 g/24-h	**189 (47.7)**	175 (40.6)	80 (25.1)	41 (15.0)	–
7–9.99 g/24-h	176 (44.4)	**199 (46.2)**	183 (57.4)	132 (48.4)	–
10–12.99 g/24-h	30 (7.6)	54 (12.5)	**52 (16.3)**	94 (34.4)	–
≥ 13 g/24-h	1 (0.3)	3 (0.7)	4 (1.3)	**6 (2.2)**	–
Misclassification	207 (52.3)	235 (54.1)	268 (83.8)	268 (97.8)	978 (68.7)
**Mage**					
< 7 g/24-h	**323 (81.6)**	319 (74.0)	182 (57.1)	112 (41.0)	–
7–9.99 g/24-h	47 (11.9)	**48 (11.1)**	76 (23.8)	72 (26.4)	–
10–12.99 g/24-h	14 (3.5)	39 (9.0)	**32 (10.0)**	36 (13.2)	–
≥ 13 g/24-h	12 (3.0)	25 (5.8)	29 (9.1)	**53 (19.4)**	–
Misclassification	73 (18.4)	386 (88.9)	288 (90.0)	221 (80.7)	968 (68.0)
**Sun**					
< 7 g/24-h	**284 (71.7)**	269 (62.4)	154 (48.3)	78 (28.6)	–
7–9.99 g/24-h	97 (24.5)	**138 (32.0)**	140 (43.9)	137 (50.2)	–
10–12.99 g/24-h	12 (3.0)	21 (4.9)	**23 (7.2)**	44 (16.1)	–
≥ 13 g/24-h	3 (0.8)	3 (0.7)	2 (0.6)	**14 (5.1)**	–
Misclassification	112 (28.3)	296 (68.2)	297 (92.8)	260 (94.9)	965 (67.8)

The numbers in bold in the table indicate the number and percentage of cases where the estimated value is consistent with the measured 24-hUNa excretion value

We also performed additional analysis to help compare our results with those of other studies. Salt intake was divided into another four groups: < 9, 9–11.99, 12–14.99, and ≥ 15 g/day. The misclassification rates of individual salt intake were almost < 55.0%, except in Kawasaki and Toft (Supplementary Table 2).

## Discussion

The evaluation method of sodium intake in this study adopted the "gold standard", that is, the 24-h urinary sodium method. In present study, we have done standardized measures and good quality control before, during and after urine collection to ensure the integrity of urine collection. The final sodium intake is basically consistent with previous studies^31^. For example, the "Report on Nutrition and Chronic Disease Status of Chinese Residents (2020)" showed that the per capita daily cooking salt in China is 9.3 g/day^32^, which is basically the same as the 9.7 g/day in present study. This study took place in the southern provinces of China, where the salt intake of residents was at a lower-middle level in the country. Meanwhile, the results of a systematic review and meta-analysis on 24-h urinary sodium excretion in China show that the sodium intake in southern China is relatively low (9.9 g/day), which is close to our research result^33^. Therefore, the estimation results of sodium intake in this study are true and reliable.

The controversy over the suitability of these formulas is obvious. Studies have shown that the original research populations when each of the eight prediction formulas were developed are different, such as gender, age, race, BMI, and whether they had high blood pressure, etc. Therefore, there are limitations in the extrapolation of the prediction formulas, and researchers may not perform population matching before conducting validation studies, resulting in poor consistency of validation results. In addition, the prediction formula uses different principles and involves different variables, which may only reflect the differences in the researchers' statistical thinking and methods.

These equations were developed through 2 major approaches: (1) direct regression of both sodium concentration from SU specimens and basic variables (such as age, height, weight, and sex) on 24-hUNa excretion and (2) multiplication of the SU sodium-to-creatinine ratio by the predicted 24-h urinary creatinine concentration. Some gender-specific, time-specific equations or those including urinary potassium concentration, but not rebuilt, were developed to estimate 24-hUNa excretion. Among the eight methods, the Kawasaki, Tanaka and INTERSALT methods are the most commonly used in clinical and epidemiologic studies. In general, the Kawasaki formula with a correlation coefficient of 0.782 between the estimated and measured 24-hUNa and the Tanaka formula with a correlation coefficient of 0.54 were developed from a healthy Japanese population, and the INTERSALT formula was developed from a natural Western population (North American and European samples). Meanwhile, the Toft formula was developed from the Danish population, the Whitton formula was developed among residents of Chinese, Malay, and Indian ethnicities in Singapore, and the Sun formula was developed based on patients with hypertension among Chinese individuals. What makes the Mage formula unique is that it was derived from NHANES research related to the collection of an SU specimen in individuals to estimate the 24-h excretion of pesticides and chemicals but not sodium. As shown in Table 1, most formulas applied urine specimens submitted at random times during the day (namely, casual spot urine), except second morning urine (SMU) was used for the Kawasaki, Whitton, and Sun formulas. The variation of these formulas could be attributable to various factors, for example, ethnicity and age structure, patterns and levels of sodium intake over the day across populations, the type of random urine used including SMU, post meridiem urine, evening urine and overnight urine, along with basic variables that are indirectly related to sodium intake, such as characteristics related to absorption and metabolism including age, gender, height, and weight. This seems to have established restriction conditions for the migration and use of different prediction formulas to external regions and populations. In addition, these prediction formulas themselves have low R-squared value (i.e. coefficient of determination) when they are developed, that will affect the deviation between the measured value and the estimated value when extrapolated.

In our study, we aimed to assess the validity of the eight predictive formulas for estimating 24-hUNa excretion by first morning urine specimens at both the population and individual levels. The results showed that no formula presented optimal performance in estimating population 24-hUNa excretion (i.e., each formula had significant biases, all p < 0.05). Except for the Kawasaki method with significant overestimation, the other seven methods significantly underestimated population 24-hUNa excretion, of which the Toft formula performed best by contrast and had the smallest bias (mean bias: − 7.9 mmol/24-h, 95% CI − 11.5 to − 4.2 mmol/24-h) with measured 24-hUNa excretion at the population level. In the present study, we also found statistically significant correlations between estimated and measured 24-hUNa excretion, but it is clear that our values of the coefficients are relatively lower than those found in other similar studies^34,35^. ICC, a more valid assessment of reproducibility and a preferable measurement of agreement, was statistically significant in our study but less than 0.320, which is within a range of agreement considered poor to fair. These results were consistent with the PURE subanalysis reporting a correlation coefficient of 0.19 to 0.29 for the Kawasaki, INTERSALT and Tanaka methods, and the ICCs ranged from 0.21 to 0.29^36^. Meanwhile, the low AUC values indicated that the formulas did not allow for the differentiation of individuals with low sodium intake artificially set from those with high sodium intake. On the other hand, the low sensitivity and specificity from the ROC curves further question the application of these formulas. It is noted that the cutoff value in the ROC curves seemed to be related to the mean measured sodium levels during the development of each formula (from Table 3 with Supplementary Table 3), which may mean that each formula has its own optimal predicted sodium range. Compared with other studies, the results of the consistency test using Bland–Altman plots (as shown in Table 3 and Fig. 2) in our study were very sufficient and provided much reliable information. Seven of eight estimates were systematically biased, with overestimation at lower levels and underestimation at higher levels, which was consistent with a meta-analysis study^37^ and with most previous studies^38^. The results showed that none of the prediction formulas has characteristics that are significantly better than the other formulas.

Regarding the verification of the validity of the prediction formulas in the Chinese population, the conclusions are also varied. Three previous studies concluded that the Tanaka formula had the least bias in predicting population 24-hUNa excretion among Chinese adults or young adolescents^24,39,40^. Nevertheless, some other studies followed up with refutation, reporting that the Kawasaki formula performed better than the Tanaka and INTERSALT formulas among natural Chinese populations or populations with hypertension^23,36,41^. There are also some beliefs that foreign forecasting formulas are not reliable^42^.

When verifying, three assumptions are often ignored, which are exactly the assumptions that the development of prediction formulas must satisfy^18,43^: (1) 24-h urine specimens were collected completely, and the factors affecting sodium excretion were excluded. Complete 24-h urine collection and evaluation is a prerequisite for the establishment and verification of the prediction formula, which requires a scientific evaluation method, and whether the urine integrity criteria used in the verification phase and formula establishment phase are consistent will also affect the results^28,44^. Meanwhile, the amount of sodium intake in the diet or sodium excretion through skin, feces, and breath exercise and urine collection time could have an effect on sodium excretion^45^. (2) The estimated creatinine excretion related to basic variables such as age, weight and height was almost equal to the actual amount of creatinine from 24-h urine specimens, and (3) the ratio of SU sodium-to-creatinine concentration was equivalent to that in 24-h urine. It is emphasized that neglecting the validation of (2) and (3) before applying the methods is likely to lead to improper use in the calculation of sodium excretion^34,46^. We calculated and displayed the data in Supplementary Table 3, which concluded that first morning fasting urine specimens in our study were acceptable for estimating 24-hUNa excretion.

The analysis results of relative and absolute differences showed that all eight methods performed poorly to estimate 24-hUNa excretion at the individual level in the current population, which was consistent with a previous study conducted among Chinese adults^39,40,42^ and with a validation study among US adults with chronic kidney disease, which pointed out that only 18–50% of individuals were stratified correctly into quartiles^47^. However, comparing Table 4 and Supplementary Table 1, the difference between which is that the cutoff point of each group has increased by 2 g/day salt, and subsequently, the misclassification rates at the individual level will fluctuate with the change of the cutoff point value. Except for Kawasaki, there was a change of more than 15% for the other seven formulas. This reminds us that the formulas should have their appropriate application range in estimating salt intake. The fact that many epidemiologic studies have classified sodium intake levels based on fixed cutoff point values of exposure or common percentile (tertile/quartile/quintile) distributions of participants’ estimated sodium excretion would induce substantial bias and have potential limitation effects^24^. The population in this study and in the Kawasaki and Tanaka study were the general Asian population, with similar ethnic characteristics, but different dietary cultures and habits led to different salt intakes. In addition, although the present study population had similar salt intake level with the INTERSALT study but had lower BMI. The Sun study was for hypertension patients, and their sodium excretion may be affected by sodium intervention and renal metabolism. It can be seen that the characteristics of the population variables during the development of the prediction formula are very important for their later extrapolation.

In general, the fact that dietary salt intake in Asian populations, especially in China, is much higher than that in their Western counterparts and the differences in dietary patterns and behaviors associated with ethnicity and culture leads to the inaccuracy of these formulas^48^. Accurate measurement of an individual’s sodium intake is essential for epidemiological studies relating sodium intake and health outcomes^49^. Some research suggested that estimated sodium intake by the formulas could change the linear relationship, while it appeared to present a J- or U-shaped relationship with mortality^38^. In fact, the prediction formula using the casual urine method for estimating daily salt intake in patients with hypertension has been compared and applied in clinical treatment^50,51^. Therefore, the deduction and application of formulas should be more cautious at the population level and at the individual level.

The novelty and strength of our study is as follows: We have a relatively large representative sample of the Chinese adult population, collect 24-h urine according to the relevant protocol to obtain high-quality urine specimens, and detect the specimens through certified quality processes to ensure the reliability of the study. Furthermore, we tested all hypotheses before formula verification. Last, this study conducted a comprehensive and informative statistical analysis, assessing the validity of the common and relative eight estimation methods at the population level by examining bias, the correlation, ICC, ROC curve and Bland–Altman plots, and at the individual level by analyzing the relative and absolute differences and misclassification, and trying to find reasons for the bias. Compared to other studies, we tested all hypotheses before formula verification. In addition, we found the reasons why various prediction formulas could not be extrapolated well, or the situations in which they were applicable. We use a variety of analysis results to explore the conditions for the promotion and use of the prediction formula, that is, as close as possible to the characteristics of the research population of the prediction formula, among which the urine sodium level may be the most important. However, the present study has some limitations, which are typical of cross-sectional population-based studies. First, a single measured concentration level of 24-hUNa excretion may not be sufficient to assess the diurnal variability of sodium excretion within person, and a single first morning fasting urine may have a lower sodium concentration in comparison with SU from other times^52^. Multiple 24-h urine collections and measurements are required to improve the precision^53^, and future studies among the Chinese population should expand to include additional spot urine specimens. Furthermore, the study was targeted for the natural population and did not adjust the factors affecting sodium absorption and metabolism, which may affect formula validation, such as the presence of mild renal dysfunction, discrepancy of the glomerular filtration rate, or recent changes in diet patterns.

## Conclusions

In conclusion, the basic characteristics of the population, such as age, sex, height and weight, involved in the development of the original prediction formula and the different ranges of urinary creatinine and sodium as a result of diet patterns and habits, led to the low reliability and validity of the extrapolation of the prediction formula. The correlation of cutoff values in the ROC curve and the corresponding grouping range with the best correct classification with the sodium intake level in the original population suggested that appropriate screening should be carried out before using the formulas. Even if the prediction formulas did not perform well at the population and individual levels in the present population, essential verification and vital statistical insights remained for the next step in the development of local formulas in China.

## Supplementary Information

Supplementary Information.

## Data Availability

The datasets analyzed during the current study are not publicly available because of intellectual property rights but are available from the corresponding author on reasonable request.

## Abbreviations

SU: Spot urine
24-hUNa: 24-Hour urinary sodium
ICC: Intraclass correlation coefficients
ROC: Receiver operating characteristic
CI: Confidence interval
INTERSALT: International Cooperative Study on Salt, Other Factors, and Blood Pressure
BP: Blood pressure
WHO: World Health Organization
CVD: Cardiovascular diseases
SRHPP: Salt Reduction and Hypertension Prevention Project
CDC: Center for Disease Control and Prevention
BMI: Body mass index
SD: Standard deviation
AUC: Area under curve
SMU: Second morning urine
