# Supplementary material for: Validity of predictive equations for 24-h urinary sodium excretion at the population and individual levels among Chinese adults aged 18–69 years
Source: Sci Rep. 2021 Nov 17;11:22404. doi: 10.1038/s41598-021-00513-1 (PMC8599737; doi:10.1038/s41598-021-00513-1)
Supplement: Supplementary file 1 — Supplementary Information. [file 41598_2021_513_MOESM1_ESM.doc]

**Online Supplemental Material**

**Validity of predictive equations for 24-hour urinary sodium excretion at the population and individual levels among Chinese adults aged 18–69 y**

hour urine collection

(n=1512)

N = 5 Counties

Filter data by criteria

**8 methods for verification:**

Kawasaki method

Tanaka method

INTERSALT1 method

INTERSALT2 method

Toft method

Whitton method

Mage method

SUN method

**Accuracy at the population level:**

Bias (Pearson’ s correlation coefficient , ICC, estimation accuracy)

Estimation accuracy (ROC, AUC, sensitivity, specificity, cutoff value)

Bland–Altman plots with linear trends and 95%CI

**Accuracy at the individual level:**

Relative difference

Absolute difference

Misclassification

Sensitivity analysis

Randomized (n=7582)

N = 5 Counties

Random selection after stratification by age and gender

Supplemental Figure 1. Study flow chart. The conclusions of this study are based on the content of the baseline survey.

Supplemental Table 1. Comparison in pairs for AUC by eight prediction formulas.

|  | Kawasaki | Tanaka | INTERSALT1 | INTERSALT2 | Toft | Whitton | Mage | Sun |
| --- | --- | --- | --- | --- | --- | --- | --- | --- |
| Kawasaki | 0 ^a^ | 0.006  (-0.001 to 0.013) | 0.000  (-0.022 to 0.022) | -0.001  (-0.022 to 0.020) | 0.031  (0.011 to 0.051)* | -0.013  (-0.036 to 0.011) | -0.015  (-0.023 to -0.007)* | -0.019  (-0.041 to 0.003) |
| Tanaka | -0.006  (-0.013 to 0.001) | 0 | -0.006  (-0.031 to 0.019) | -0.007  (-0.031 to 0.016) | 0.025  (0.000 to 0.050)* | -0.019  (-0.046 to 0.009) | -0.022  (-0.029 to -0.014)* | -0.026  (-0.050 to -0.001)* |
| INTERSALT1 | 0.000  (-0.022 to 0.022) | 0.006  (-0.019 to 0.031) | 0 | -0.001  (-0.003 to 0.001) | 0.031  (0.016 to 0.045)* | -0.013  (-0.030 to 0.004) | -0.015  (-0.038 to 0.007) | -0.019  (-0.042 to 0.003) |
| INTERSALT2 | 0.001  (-0.020 to 0.022) | 0.007  (-0.016 to 0.031) | 0.001  (-0.001 to 0.003) | 0 | 0.032  (0.018 to 0.046)* | -0.012  (-0.029 to 0.006) | -0.014  (-0.036 to 0.008) | -0.018  (-0.040 to 0.004) |
| Toft | -0.031  (-0.051 to -0.011)* | -0.025  (-0.050 to 0.000)* | -0.031  (-0.045 to -0.016)* | -0.032  (-0.046 to -0.018)* | 0 | -0.043  (-0.062 to -0.025)* | -0.046  (-0.070 to -0.023)* | -0.050  (-0.071 to -0.030)* |
| Whitton | 0.013  (-0.011 to 0.036) | 0.019  (-0.009 to 0.046) | 0.013  (-0.004 to 0.030) | 0.012  (-0.006 to 0.029) | 0.043  (0.025 to 0.062)* | 0 | -0.003  (-0.027 to 0.022) | -0.007  (-0.027 to 0.014) |
| Mage | 0.015  (0.007 to 0.023)* | 0.022  (0.014 to 0.029)* | 0.015  (-0.007 to 0.038) | 0.014  (-0.008 to 0.036) | 0.046  (0.023 to 0.070)* | 0.003  (-0.022 to 0.027) | 0 | -0.004  (-0.023 to 0.015) |
| Sun | 0.019  (-0.003 to 0.041) | 0.026  (0.001 to 0.050)* | 0.019  (-0.003 to 0.042) | 0.018  (-0.004 to 0.040) | 0.050  (0.030 to 0.071)* | 0.007  (-0.014 to 0.027) | 0.004  (-0.015 to 0.023) | 0 |

^a^ The difference (95%CI) is the estimated value in the row minus the estimated value in the column.

* *p* < 0.05.

Supplemental Table 2. Misclassification of the eight predicted methods for individual salt intake level, n(%).

| Method | Conversion of Salt Intake by 24-hUNa Excretion | | | | Total  (n = 1424) |
| --- | --- | --- | --- | --- | --- |
|  | < 9 g/24-hour (n = 690) | 9 ~ 11.99 g/24-hour (n = 367) | 12 ~ 14.99 g/24-hour (n = 206) | ≥15 g/24-hour (n = 161) |  |
| Kawasaki | 392 (56.8) | 226 (61.6) | 144 (69.9) | 121 (75.2) | 883 (62.0) |
| Tanaka | 157 (22.8) | 254 (69.2) | 191 (92.7) | 157 (97.5) | 759 (53.3) |
| INTERSALT1 | 121 (17.5) | 260 (70.8) | 196 (95.1) | 161 (100.0) | 738 (51.8) |
| INTERSALT2 | 97 (14.1) | 275 (74.9) | 198 (96.1) | 161 (100.0) | 731 (51.3) |
| Toft | 232 (33.6) | 254 (69.2) | 173 (84.0) | 152 (94.4) | 811 (57.0) |
| Whitton | 133 (19.3) | 248 (67.6) | 199 (96.6) | 160 (99.4) | 740 (52.0) |
| Mage | 89 (12.9) | 323 (88.0) | 192 (93.2) | 140 (87.0) | 744 (52.2) |
| Sun | 59 (8.6) | 328 (89.4) | 202 (98.1) | 158 (98.1) | 747 (52.5) |

Supplemental Table 3. Verification of related hypotheses (n = 1424).

| Method | Original value of the test data sets in the published literature | | | | |  | Predicted value in this study |  | Bias | | |
| --- | --- | --- | --- | --- | --- | --- | --- | --- | --- | --- | --- |
|  | Age(years) | BMI (kg/m^2^) | Mean measured 24-hourUNa (mmol/24-hour) | Mean measured 24-hour urinary creatinine (mg/24-hour) | Correlation coefficient (r_p_) between the ratio of spot SU sodium-to-creatinine concentration and that in 24-hour urine |  | Mean estimated 24-hour urinary creatinine (mg/24-hour) |  | Mean creatinine Bias (mg/24-hour, 95%CI) ^a^ | r_p_ ^b^ | ICC (95%CI) ^c^ |
| Kawasaki | 34.0 ± 1.2 | 22.2 ± 0.3 | 209.1 ± 6.8 | 1322.5 ± 18.9 | 0.774 |  | 1289.1 ± 363.3 |  | 211.4 (188.1 to 234.7) | 0.393 | 0.34 (0.21 to 0.45) |
| Tanaka | 39.5 ± 11.2 | 22.4 ± 2.9 | 187.2 ± 65.0 | 1122.7 ± 210.3 | 0.650 |  | 1205.6 ± 266.4 |  | 128.0 (105.4 to 150.6) | 0.325 | 0.27 (0.21 to 0.33) |
| INTERSALT1 | - | 25.8 ± 4.0 ^d^ | 163.0 ± 37.1 | - | NA |  | NA |  | NA | NA | NA |
| INTERSALT2 | - | 25.8 ± 4.0 | 163.0 ± 37.1 | - | NA |  | NA |  | NA | NA | NA |
| Toft | - | - | 150.0 (64.0 to 305.0)^e^ | 1223.0 (715.0 to 2241.0)^e^ | - |  | 1309.7 ± 339.8 |  | 232.1 (209.4 to 254.7) | 0.400 | 0.33 (0.17 to 0.46) |
| Whitton | 49.4 ± 14.9 | 24.0 ± 4.7 | 125.0 ± 53.4 | - | NA |  | NA |  | NA | NA | NA |
| Mage | NA | NA | NA | NA | NA |  | 1034.3 ± 366.4 |  | -43.3 (-67.9 to -18.8) | 0.325 | 0.32 (0.27 to 0.36) |
| Sun | 58.4 ± 14.5 | 25.9 ± 3.5 | 147.9 ± 61.8 | 1249.2 ± 61.1 | 0.507 |  | 1305.8 ± 315.0 |  | 228.1 (204.9 to 251.4) | 0.336 | 0.27 (0.14 to 0.38) |

^a^ Mean measured 24-hour urinary creatinine was 1077.6±440.7 mg/24-hour in the present study. Bia equals estimated -measured value. Mean bias all p <0.001.

^b^ Person correlation all *p* < 0.001.

^c^ The value of single measures were used , and all *p* < 0.001.

^d^ This data is the mean of the test set and validation set listed in the published literature.

^e^ Values are median (5th percentile to 95th percentile) listed in the published literature.

NA in Mage indicates that there is not data applied on participant samples in the method. NA in INTERSALT and Whitton, indicates that these formulas perform direct regression of both sodium concentration from SU specimens and basic variables on 24-hUNa excretion, and do not need these data. '-' in INTERSALT and Toft, indicates there is no relevant data listed in the published literature.

24-hUNa, 24-hour urinary sodium, r_p_, person correlation coefficient, ICC, intraclass correlation coefficients.
